# Impact of timing of soft tissue augmentation on the width of keratinized mucosa around the dental implant

**DOI:** 10.1007/s10006-025-01401-y

**Published:** 2025-05-20

**Authors:** Mohamed Ahmed Hafez El-Sayed, Wafaa Saleh, Samah ElMeadawy, Mohamed A. Al-Shahat

**Affiliations:** Oral Medicine, Periodontology, Diagnosis and Oral Radiology Department, Faculty of Dentistry, Mansoura University, Mansoura, 33516 Egypt

**Keywords:** Denal implant, Free gingival graft, Augmentation

## Abstract

**Purpose:**

This study measures the impact of timing of free gingival graft (FGG) in improving aesthetics, function, and long-term stability of dental implants by measuring keratinized mucosa width (KMW), soft tissue thickness (STT), and graft shrinkage after implant treatment.

**Methods:**

The current randomized clinical trial included 20 patients with inadequate KMW and requiring placement of dental implant. The participants were randomly distributed into two groups. Group 1 received FGG 2 months before placing the dental implant while group 2 received the FGG at the second stage of implant surgery. The following parameters were evaluated and statistically analyzed at baseline, day 7, as well as 1, 3, 6, and 9 months postoperatively, KMW, STT and shrinkage percentage of the FGG.

**Results:**

Twenty patients with ages ranging from 30 to 55 years with reduced KMW were included in the current study. Both groups showed a significant increase in KMW and STT on day7, 1, 3, 6, and 9 months postoperatively while the intergroup comparison showed no significant differences in KWM, STT, and shrinkage percentage between both groups at the same point of time.

**Conclusion:**

The two study groups’ outcomes demonstrated that the FGG can be placed either before or after dental implants to improve the soft tissue augmentation surrounding the implants. However, the implantologist should take patient preferences and long-term stability into consideration.

## Introduction

Dental implant surgery is the process of replacement of missed teeth with artificial teeth to restore the function and esthetics of natural teeth [1]. Nowadays, the awareness of most implantologists has moved from only achieving osseointegration to additionally the success of an attractive aesthetic appearance. As a result, there was a growing interest in the augmentation of soft tissue around dental implants [2]. Recognizing the growing significance of keratinized tissue around dental implants for maintaining tissue stability is important to implant success. Therefore, managing soft tissue becomes an important factor in achieving aesthetically pleasing implant restoration [3]. 

There is a developing importance on the necessity of keratinized tissue around dental implants to enhance tissue stability and health. In the early years of implant dentistry, only a limited number of comparative studies examined the correlation between peri-implant tissue health and the width of keratinized mucosa [4]. The absence of keratinized tissue around implants has been linked to increased plaque accumulation, gingival inflammation, greater mucosal recession, and reduced esthetic appearance [5]. Broader bands of keratinized mucosa show marked reductions in attachment loss, plaque accumulation, mucosal recession, and gingivitis. Additionally, there are obvious shifts towards shallower probing depths and diminished radiographic bone loss [6, 7]. 

Keratinized mucosa width (KMW) and soft tissue thickness (STT) stand out as critical factors influencing the aesthetics, function, and stability of implants [8]. In cases where KMW is insufficient (< 2 mm), a significant correlation exists with the severity of peri-implant mucositis [9]. The stability of the marginal bone around the dental implant is strongly affected by STT at the crest of a dental implant. It has been reported that cases of thin gingival biotype experienced a crestal bone loss of up to 1.45 mm, despite the supra-crestal position of the implant-abutment interface [10]. 

Clinicians have applied various techniques to augment gingival tissue. These techniques comprise the application of diverse autograft, allograft, or xenograft materials [11–13]. The application of these techniques can augment soft tissue volume both during implant placement and/or the second-stage surgery. Techniques include Free gingival graft (FGG), Subepithelial Connective Tissue Graft, Connective tissue graft, acellular dermal matrix combined with an envelope flap or pouch, Platelet-rich fibrin, and Pedicle grafts [14–16]. 

Traditionally regarded as the gold standard for peri-implant soft tissue augmentation, autogenous soft tissue grafts, such as FGG, can be obtained from the highly keratinized hard palate. However, challenges persist in accomplishing a natural blend of color and shade with the surrounding soft tissues [17]. 

Soft tissue augmentation around dental implants can be employed at different stages during the implant treatment, including phases before, after, or even during the loading of dental implants [18]. There is a debate in the available literature about the influence of time on the results of soft tissue augmentation. Consequently, this study aims to carefully assess how the timing of peri-implant soft tissue augmentation affects the overall results from the perspective of peri-implant soft tissue.

## Methods

The current study was conducted at the Periodontology clinic, Department of Oral Medicine and Periodontology, Faculty of Dentistry, Mansoura University. The participants were selected from the patients with missing teeth and attending clinic to receive dental implants. The study design was approved by the Ethical Committee at the Faculty of Dentistry, Mansoura University (Reference number: A11100221), and this clinical trial was registered at ClinicalTrials.gov (Identifier: NCT06238427). Written informed consent was obtained from all participants before their involvement in the study. This study was conducted following the Helsinki Declaration for the ethical principles for research involving human subjects.

### Patients’ selection

We carefully selected the participants of the current study according to the following criteria. We included patients requiring dental implants with KMW less than 2 mm at the proposed implant site, and patients who were aged 21 years or older with no systemic condition that affects the implant or grafting surgery. In addition, the following criteria were required; a buccolingual bone width of at least 5.5 mm, a mesiodistal distance of at least 7 mm between adjacent teeth in a single edentulous space, a minimal bone height not less than 10 mm, a well-formed residual alveolar ridge covered with firm mucosa, absence of signs of periodontal bone loss or significant soft tissue loss in teeth adjacent to the implant site, and adequate inter-arch space exceeding 7 mm.

The exclusion criteria were employed to exclude uncooperative patients, Patients with abnormal parafunctional habits, e.g., bruxism and clenching, and Patients who have a systemic or local illness that would disrupt healing. Patients receiving systemic corticosteroids or any other drug that may affect osseointegration or post-operative healing. In addition, smokers with more than 10 cigarettes/day were excluded.

### Random distribution of the participants

The participants were randomly allocated into two groups using computer generated list of randomizations with a randomization table using SPSS V23.0. Both groups received submerged dental implant protocol and FGG. Group 1 received FGG 2 months before placing the dental implant while group 2 received the FGG at the phase of second stage implant surgery.

In addition, each patient received a number from the randomization list, and it was placed in a sealed opaque envelope while the participants were blinded by the treatment protocol and the envelope was opened immediately before surgery. A single-blinded outcome assessor performed all the periodontal and radiographic assessments. This assessor was blinded by the treatment protocol and the allocation of patients into the two groups.

### Preoperative patient assessment

Complete medical and dental histories were recorded for all patients and complete clinical intraoral and extraoral examinations were performed. Selected patients were questioned about the cause and time of extractions, whether extractions were because of periodontal disease, dental decay, or other causes. Previous experiences with dental procedures were also discussed. Intraoral photographs were taken to record the existing condition of teeth and mucosa before implant placement. The density and bone levels at the implant site were assessed by CBCT. Consequently, the proper implant size was selected and placed in the ideal position.

At baseline, periodontal and gingival conditions were evaluated for each patient including the following parameters: KMW and STT. The KMW was measured from the zenith of the alveolar ridge to the muco-gingival junction (MGJ) using a calibrated periodontal probe. MGJ was located using a functional test which is a reproducible method for keratinized gingival width assessment by stretching the lip and cheek or by placing a probe horizontally in the vestibule and rolling to the mucosa coronally, the MGJ is where the mucosa stops rolling or moving.

STT was determined 1.5 mm apical to the soft tissue margin (STM) with a short anesthesia needle or endodontic micro-opener with a silicon stopper. The needle was inserted perpendicular to the mucosal surface, through the soft tissues with light pressure until a hard surface. The silicon stopper was then placed in tight contact with the soft tissue surface with its coronal border overlapping the STM. Then the penetrated part was measured on the endodontic ruler and measurements were rounded to the nearest half of a millimeter.

### Intervention procedures

#### First stage surgery

All the surgical procedures were performed by the same periodontist to standardize the procedures. Before starting the surgery, patients were asked to rinse their mouths with chlorhexidine gluconate for 1 to 2 min to decrease the bacterial load around the surgical site.

After administration of local anesthesia, a mesiodistal mid-crestal incision was made at the proposed implant site where a full mucoperiosteal flap was elevated to the MGJ buccally and lingually. After that, a large round bur was used to recontour the narrow ridge and remove the granulation tissue from the bone in the implant site. Drilling was started carefully to calculate the depth for each implant using the pilot drill. A periapical x-ray was done with the pilot drill in place to evaluate the direction and depth of the drilling in relation to the roots of the neighboring teeth and to vital structures. A parallel pin was placed in the prepared hole to check the alignment and parallelism of future implant position in relation to the neighboring teeth. Sequential drilling was then continued to prepare the site according to the selected implant size. Copious irrigation with cold saline was done during the surgical procedure to avoid overheating of bone which causes bone necrosis and increases the risk of failure. The used implant was an acid etched, tapered, with internal helix connection titanium dental implant (Neobiotech IS II active dental implant).

A torque wrench and an insertion tool were used to insert the implant. A torque of at least 35 to 40 N-cm was obtained, ensuring the implant’s initial stability. The implant was installed at the site of the osteotomy, submerged under the margin of the crest by 1 mm. The cover screw was screwed to the implant, the surgical site was thoroughly irrigated with sterile saline to remove debris and clean the wound and the flap was approximated and sutured using 5\0 monofilament suture.

#### Second stage surgery

The second stage of surgery was conducted 2 to 4 months after placement of the implant. Local anesthesia was administered, followed by an incision for the exposure of the cover screw. Any hard tissue overgrowth was removed using a small rose head bur, and the cover screw was taken out. Then, the solid non-customized healing abutment of suitable size and length was connected to allow its emergence through the soft tissues, which was confirmed by a periapical X-ray. Suturing around the healing abutment was performed using a 4/0 non-resorbable suture. The patients were prescribed mouthwash, analgesics, and oral hygiene measures.

#### Recipient site preparation for FGG

In group 1, the recipient site was prepared 2 months prior to placing the implant. A horizontal incision was performed at the MGJ level along the recipient area’s length. Vertical releasing incisions, slightly divergent toward the sulcus depth, were performed at both mesial and distal ends. Utilizing scalpel 15c, a partial-thickness flap was raised, preserving the nonmobile, firm periosteum connected to the underlying bone to maintain blood supply to the graft. The repositioned flap created space for the graft, free from muscle tension. Careful suturing with 6/0 non-resorbable suture (Proline, Ethicon, USA) stabilized the borders and base, and tin foil paper was trimmed to the graft’s size and validated at the recipient site.

**Fig. 1:**
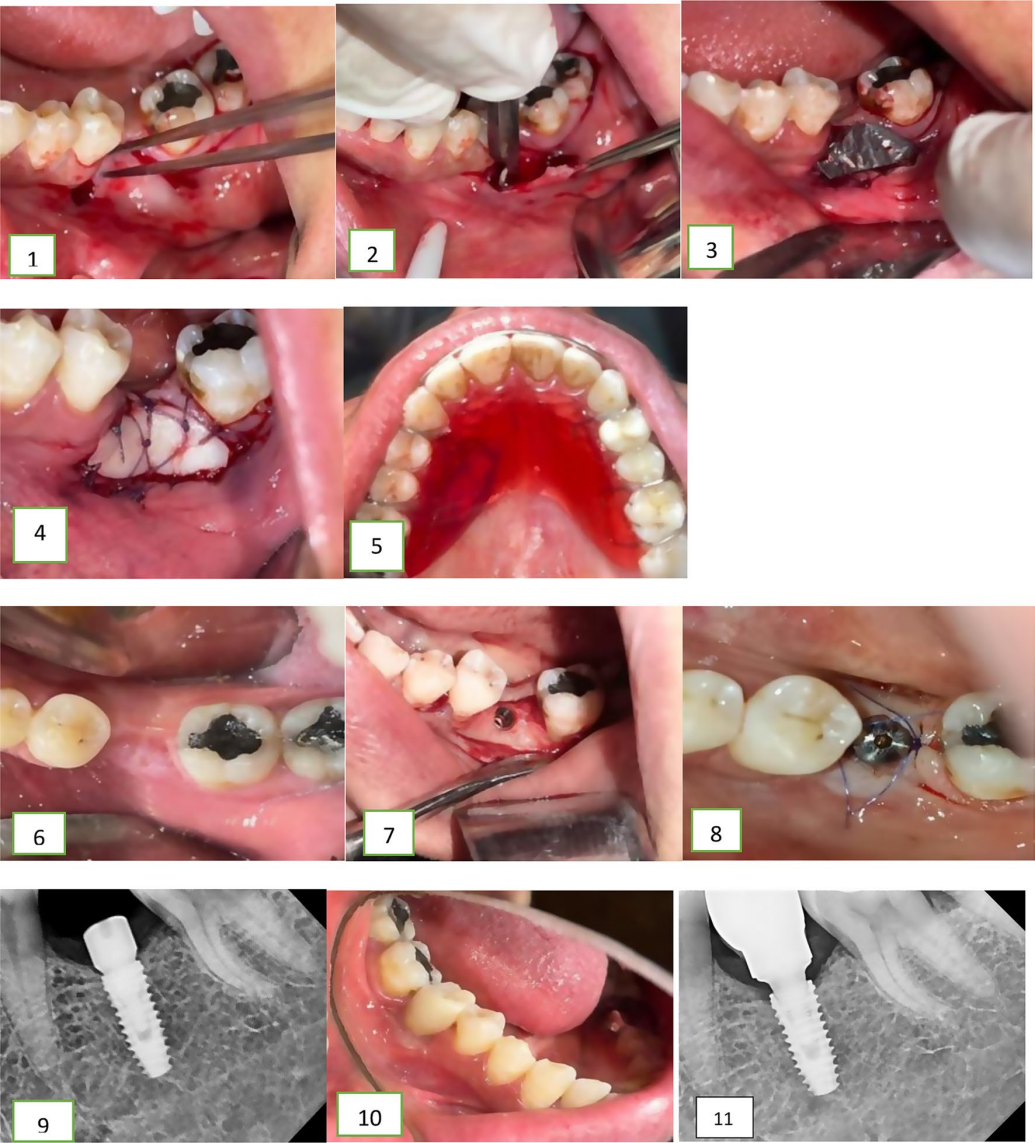
Photographs of case from group 1 receiving FGG 2 months prior to implant placement. This graph shows: (1) Horizontal incision along MGJ (2) Partial thickness flap elevation (3) Tin foil adjustment on the recipient site (4) The graft was sutured in recipient site. (5) Acrylic stent in place. (6) Initial healing of the graft (7) Implant installation in the osteotomy site. (8) Healing abutment installation in the 2nd stage surgery, 3 months after implant placement. (9) Periapical x ray with healing abutment in place. (10) Final prosthesis

**Fig. 2:**
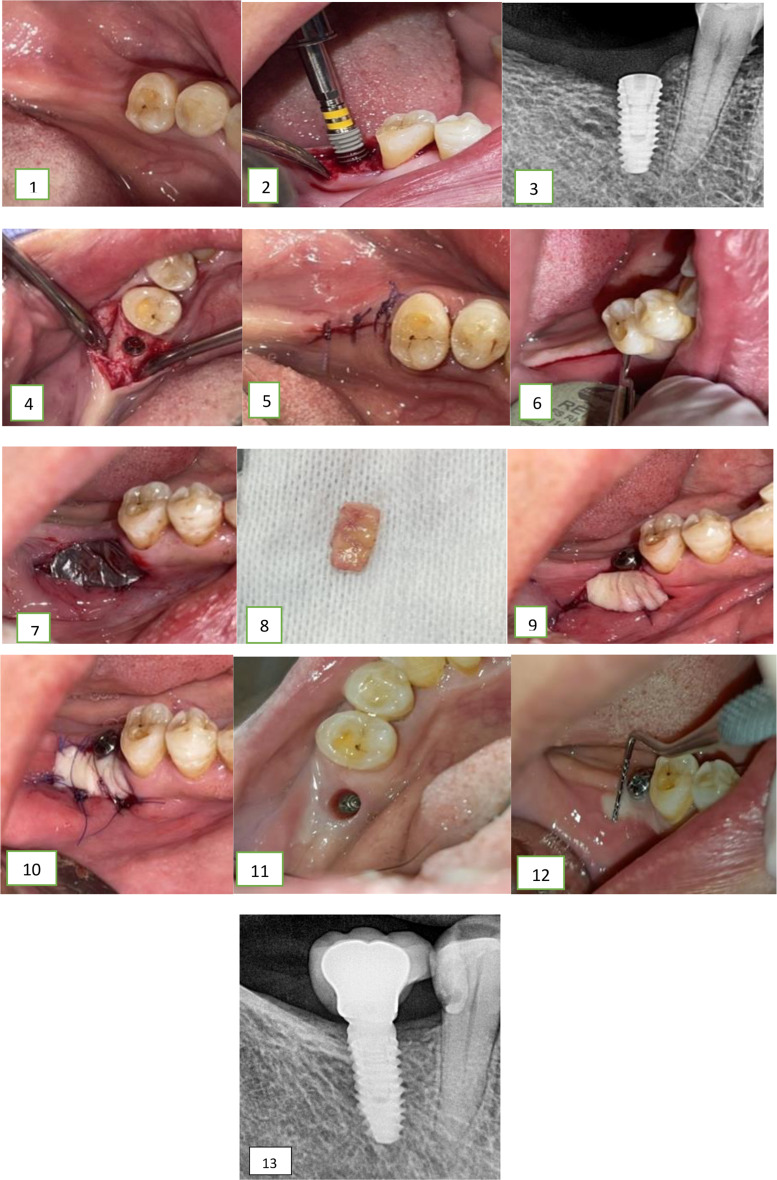
Graphs for a case from group 2 with FGG done at time of second stage surgery of dental implant. These graphs show: (1) Preoperative view of the proposed implant site (2) Implant installation with hand wrench (3). Periapical x ray after implant installation. (4) The implant in the osteotomy site. (5) Sutured flap site. (6) Horizontal incision along MGJ. (7) Tin foil adjustment on the recipient site. (8) The FGG harvested from the palate. (9) The graft was adapted in the recipient site. (10) The graft was sutured. (11) Emergence profile. (12) Assessment of KTW gain

In group 2, the horizontal incision was done at the second surgical stage. It was incised along the length of the recipient area at the MGJ. Subsequent steps in grafting were performed similarly to Group 1. Using a sharp blade, tissues were dissected sharply lingual to the incision line ensuring thorough cleaning of the implant head. Any hard tissue overgrowth was removed using a small round bur. Following the connection of the healing abutment, a periapical X-ray film was taken to verify its complete seating (Figs. 1 and 2).

#### FGG harvesting

**Fig. 3:**
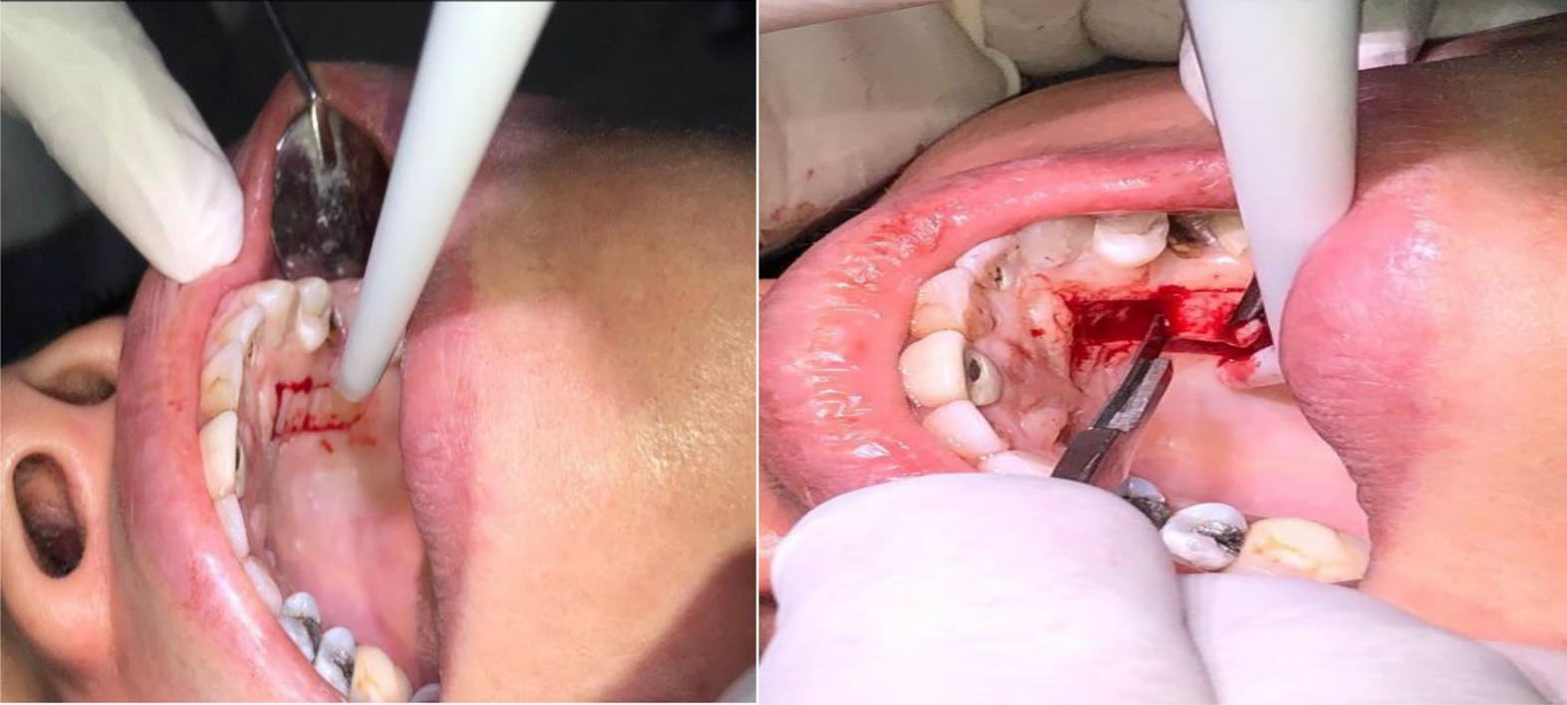
Harvesting the FGG from the palate

The FGG was harvested from the hard palate using a tinfoil template in the area bounded by the canine and the maxillary first molar. The depth of the incision was 2 mm. The graft’s edge was gently elevated by tissue forceps and the scalpel was used to carefully detach the graft from the palate (Fig. 3).

The graft was subsequently immersed in isotonic saline and placed on a wet sterile gauze. A surgical gel foam sponge was applied to enhance hemostasis. Then the surgical site was covered with a prefabricated acrylic palatal stent. Suturing was achieved with 6/0 non-resorbable proline sutures. Following the suturing process, finger pressure was applied using wet sterile gauze over the graft for five minutes. This was done to ensure intimate contact and eliminate any potential dead space between the graft and the vascular bed of the recipient site (Figs. 1 and 2).

### Prosthetic phase

Indirect impression for the implants was performed by closed tray impression technique as follows: Healing abutments were unscrewed from the implants and impression transfers were installed instead. The impression was taken with elastomeric impression material and closed tray impression technique. After material setting, the impression was removed from the oral cavity, impression-transfer unscrewed from the implant and the healing abutment applied back in place.

Impression transfers were connected to the implant analog to form one unit assembly then the assembled components were seated carefully to their representative place in the impression.

The impression was sent to the lab with a suitable prosthetic abutment for crown fabrication. Then the crown was checked and cemented using glass ionomer cement.

### Clinical assessment

KMW, STT, and graft shrinkage were assessed at the following times: baseline, after initial healing of the FGG (7 days after FGG), one month after FGG, 3 months after FGG i.e. one month after implant placement (for group1), one month after implant loading (for group2), 6 months after FGG i.e. one month after implant loading for (group1) and four months after implant loading for (group2) and nine months after FGG i.e. four months after implant loading for (group1) and seven months after implant loading for (group2).

The shrinkage percentage of the gingival graft was measured at 1, 3, 6, and 9 months after the initial healing of the graft. First, the surface area of the graft was determined by multiplying the length by the width. All the measurements at follow-up points of time were compared to the measurement after the initial healing to calculate the shrinkage percentage [19]. 

### Radiographic assessment

After the implant placement in group 1 and 3 months after the second stage surgery in group 2, the radiographic assessment was performed using periapical X-ray with paralleling technique to evaluate the bone level around the dental implant. Analysis of the X-ray was done by the Clear Dent program to measure the distance between the implant collar and the bone level.

### Sample size calculation and statistical analysis

The sample size was established using the null hypothesis, which stated that the results of the first and the second groups were not equal. The desired study power was 95% with a confidence interval of 95%. 18 patients were deemed to be the necessary sample size using the G power software (version 3.1.9). The study’s sample size was raised to 20 patients (10 in each group) to account for potential candidate attrition without the potential for altered results. Data calibration was performed before the start of the study to ensure the reliability of the measurements. Sample analysis involved evaluation of clinical and radiographic outcomes at different follow-up times.

Data entry and statistical analyses were done using SPSS version 16.0 (Inc., Chicago, IL, USA). Data were first tested by Shapiro-Wilk test for distribution of data. The mean and standard deviation were used to express parametric data. An Independent t-test was done to compare two means in the study groups. The statistical analysis was performed based on significance level of 0.05 alpha error to reject the null hypothesis.

## Results

Twenty eligible patients were included in the current randomized clinical trial. The age of the participants in both groups ranged from 30 to 55 years old. Group 1 received FGG 2 months before placement of the implant while participants in group 2 received the FGG during the second stage surgery of the implant.

We evaluated the KMW at baseline and different follow-up times in both groups. We found that both groups had a significant increase in KMW at day 7 as well as 1, 3, 6, and 9 months (*P* < 0.00001) with no significant difference between both groups at the same point in time. Moreover, the KMW decreased over time in both groups with no statistically significant differences. The STT showed a statistically significant increase from baseline to all the follow-up times in both groups including days 7 and 1, 3, 6, and 9 months after surgery (P1 < 0.00001). Moreover, stability of STT was noticed in both groups at 1, 3, 6, and 9 months postoperatively with no significant differences in STT at those follow-up times (*P* > 0.05).

Table 1 shows the mean shrinkage percentage after one, 3, 6, and 9 months of follow-up for both groups. Group 1 and Group 2 didn’t show a significant change in the shrinkage percentage of the FGG at different follow-up times. In addition, no difference was detected between both groups at 1, 3, 6, and 9 months follow-up (*p* > 0.05).

As regards the effect of implant loading on graft stability, the shrinkage percentage of grafts of group 1 was not statistically significantly different from that of group 2 at 1 and 4 months of implant loading (Table 2).

**Table 1:** Evaluation of clinical parameters between the two groups at different points of time

	Group 1	Group 2	*P*-value comparison between groups			
			Group1		Group2	
**Keratinized mucosa width (KMW)**						
Baseline (mean ± SD)	0.75 ± 0.26	0.9 ± 0.42	P1 < 0.00001* p2 < 0.00001* p3 < 0.00001* p4 < 0.00001* p5 < 0.00001* p6 = 0.12 p7 = 0.58 p8 = 0.3	P1 < 0.00001* p2 < 0.00001* p3 < 0.00001* p4 < 0.00001* p5 < 0.00001* p6 = 0.05* p7 = 0.58 p8 = 0.3		
Day7 after surgery(mean$\:\pm\:$SD)	8.2 ± 0.75	8.6±$\:\:0.51$				
1 month (mean ± SD)	6.8 ± 0.67	7.4$\:\:\pm\:0.42$				
3 months (mean ± SD)	6.35 ± 0.62	7.04 ± 0.43				
6 months (mean ± SD)	6.2 ± 0.58	6.96 ± 0.68				
9 months (mean ± SD)	5.95 ± 0.47	6.52 ± 0.51				
**Soft tissue thickness (STT)**						
Baseline (mean ± SD)	1.05 ± 0.43	0.8 $\:\pm\:$ 0.35	P1 < 0.00001* p2 < 0.00001* p3 < 0.00001* p4 < 0.00001* p5 < 0.00001* P6 = 0.3 P7 = 0.438 P8 = 0.17	P1 < 0.00001* p2 < 0.00001* p3 < 0.00001* p4 < 0.00001* p5 < 0.00001* P6 = 0.08 P7 = 0.6 P8_=_ 0.3		
Day7 after surgery (mean ± SD)	3 ± 0.33	2.70$\:\pm\:$0.42				
1 month (mean ± SD)	2.55 ± 0.58	2.55 ± 0.36				
3 months (mean ± SD)	2.4 ± 0.31	2.35 ± 0.24				
6 months (mean ± SD)	2.3 ± 0.25	2.31$\:\pm\:$0.22				
9 months (mean ± SD)	2.15 ± 0.24	2.2$\:\pm\:$0.22				
**Shrinkage % of the graft**						
1 month (mean ± SD)	13.98 ± 0.66	13.98 ± 0.66	*P* = 0.36	P6 = 0.064 P7 = 0.20 P8 = 0.10		P6 = 0.247 P7 = 0.244 P8 = 0.277
3 months (mean ± SD)	14.36 ± 0.64	14.2 ± 1.14	*P* = 0.35			
6 months (mean ± SD)	14.6 ± 0.6	14.59 ± 1.31	*P* = 0.49			
9 months (mean ± SD)	14.96 ± 0.61	14.94 ± 1.28	*P* = 0.48			

P: p value for comparing the two groups at the same time

p1: p value for comparing day 7 after surgery and baseline of the same group

p2: p value for comparing After 1 month and baseline of the same group

p3: p value for comparing between 3months postoperative and baseline of the same group

p4: p value for comparing between 6 months postoperative and baseline of the same group

p5: p value for comparing between 9 months postoperative and baseline. of the same group

p6: p value for comparing between 1 month and 3 months postoperative of the same group

p7: p value for comparing between 3 months and 6 months postoperative of the same group

P8: p value for comparing between 6 month and 9 months postoperative of the same group

*: Statistically significant at *p* ≤ 0.05

**Table 2:** Comparison of shrinkage percentage between studied groups after 1 and 4 months of implant loading:

	Group 1	Group 2	Test of significance
Shrinkage % mean ± SD after 1 month of implant loading	16.32$\:\pm\:$4.26	15.51 ± 3.29	t = 0.3711 *p* = 0.593
Shrinkage % mean ± SD after 4 months of implant loading	17.54$\:\pm\:$3.87	17.41$\:\pm\:$3.18	t = 0.518 *p* = 0.6107

t: Student t test,

*: Statistically significant if *p* < 0.05

## Discussion

Adequate keratinized mucosa is essential for decreasing peri-implantitis and enhancing the long-term stability of the dental implant. Our randomized clinical trial specifically investigated the impact of the timing of soft tissue grafts during implant therapy, aiming to reveal its impact on the stability of peri-implant soft tissue [20]. 

There is a debate concerning the optimal timing for gingival tissue augmentation around the dental implant. The management of soft tissue around dental implants suggests various time points for soft tissue grafting, including before implant placement, concurrently with implant placement, during the second stage surgery of the implant, or following the fitting of the final restoration [21]. 

In our study, we selected two separate time intervals for free gingiva grafting: In group 1, FGG was executed two months before implant placement, and in group 2, FGG was done during the second stage surgery, in agreement with the recommendation by Kadkhodazadeh et al., who employed various treatment groups with soft tissue grafting at different times [18]. 

The rationale of soft tissue graft before implant placement is that implant surgery is done in previously mature augmented tissues which are easily handled. This approach may enhance the blood supply to the tissues with tension-free flap coverage as well as increase the overall stability of the tissues. Furthermore, it is a less complicated technique, as each of the grafting steps and implant placement are completed in separate surgeries [22]. 

On the other hand, performing the soft tissue graft during the second stage surgery of implant involves a fewer number of surgeries and consequences in a quicker healing with less pain and discomfort. In addition, it decreases costs and improves overall patient satisfaction [18]. Nevertheless, it is a more delicate technique that requires more experience and may increase the patient’s discomfort because of long time surgery [23]. 

The soft tissue augmentation in our study was done by the combination of FGG and vestibuloplasty with the apically positioned flap to increase the keratinized tissue dimensions [24].The application of the FGG was designed to improve the tissues’ regeneration, due to its packed cell content, and it is the optimal treatment option to enhance the KTW, STT, long-term graft stability, and an increased success rate. Remarkably, this technique is characterized by a high level of success and doesn’t necessitates high cost due to obtaining the graft from the patient’s own oral cavity [25]. 

However, obtaining FGG from the palate is associated with certain complications including morbidity of the donor site and limited amount of tissue obtained. In addition, an alteration in the color and texture of the grafted tissue was noticed if compared to the adjacent recipient tissues. Furthermore, this technique is associated with extended surgical time and healing process [26]. 

Regarding the KMW in our study, we observed that both groups experienced a statistically significant increase of KMW at the different follow-up times while the intergroup analysis revealed no significant differences between the two groups at the same time of follow-up. We noticed stability in KMW in both groups after the initial healing of the FGG without a major difference in KMW between those periods.

An earlier published literature focused on peri-implant KMW in different studies, grouping them based on baseline data into two categories: ≥2 mm and < 2 mm. From baseline to over 1 year, the mean KMW change was 0.55 mm in the ≥ 2 mm group and 2.56 mm in the < 2 mm group, with an overall mean of 1.69 mm. Considering timing, KMW showed more change in the < 2 mm group, but similar values in simultaneous and staged groups. A comparison at 3 months and > 3-month healing showed a statistically non-significant difference in KTW gain (weighted mean difference: 0.21, 95% CI: 0.13–0.55) [27]. Other studies that compared KMW values after three months and beyond three months of healing also support this finding. While there was an increase in KMW at three months as opposed to after three months, no statistically significant difference was seen in these investigations [28–30]. 

In addition, this study showed a significant improvement in STT after the positioning of a 1.5 mm FGG upon initial healing (7 days after surgery) compared to baseline. Though, a minor statistically significant, post-operative swelling was noted during removal of the suture. This may be attributed to the trauma of the vertical releasing incisions, resulting in postoperative edema. The gain in STT gradually decreased during follow-up times. The substantial gain in STT accomplished after FGG augmentation in both groups compared to baseline agreed with other studies, suggesting that soft tissue grafting during implant treatment enhances the contour and aesthetics [27]. 

The current literature analyzed the impact of soft tissue graft timing on the STT in which soft tissue augmentation was performed. The augmentation of the soft tissue was done 3 to 6 months after implant placement or at stage 2 surgery. The authors found no significant difference in STT between both groups. In addition, the analysis revealed that both groups experienced most of their shrinkage percentage during the first month of follow-up, with no obvious difference between them [27]. In addition, a controlled clinical study conducted by Schmitt et al. [31] reported a KTW shrinkage of 14.59% at 30 days after FGG, coinciding with our results.

We examined the effect of implant loading on graft stability. We didn’t find a significant change in the graft shrinkage after the implant loading in both groups and within the same group. The implication is that soft tissue augmentation is carried out at two distinct times—two months before implant implantation and during the second stage to produce stable tissue that is not negatively impacted by implant loading. Our findings align with controlled clinical studies conducted by Schmitt et al. 2013 [29] where KMW was measured at the time of impression taking for final prosthesis fabrication as the baseline to assess KMW shrinkage after loading. Longer follow-ups (≥ 12 months) demonstrated no further shrinkage of KMW at the buccal aspect, indicating relative stability over time.

In addition, the results of our study showed (0.17 mm) of mesial bone loss for both groups and (0.168 and 0.148 mm) of distal bone loss for group 1 and group 2 respectively 6 months after implant placement with no significant difference between both groups. These results are in agreement with the results of a previous study that detected (0.18 and 0.44 mm) mean bone loss in a group with keratinized mucosa width ≥ 2 mm and a group with keratinized mucosa width < 2 mm respectively that reveals a significant difference between both groups [32]. Our explanation for this result is that using FGG provides better soft tissue seal around implants and decreases the possibility of peri-implantitis which is the major cause of crestal bone loss around implants [33]. 

In conclusion, the current randomized clinical trial showed that both groups showed significant improvements in KTW and STT while group 1 procedures are easy to conduct and the implant placement was performed in an already augmented tissue. However, it requires second surgery for grafting. On the other hand, group 2 procedures were time saving with a fewer number of surgeries with rapid healing and more patient satisfaction.

### Limitations of the study

Despite the valuable outcomes of the current study, we acknowledge certain limitations. First, our study included only 20 patients which is considered a small sample size. Second, we recommend a longer follow-up duration up to five years to detect the changes over time with a more comprehensive understanding of treatment outcomes. Hence, we recommend further randomized clinical trials with larger sample size and longer follow-up periods.

## Data Availability

The data that support the findings of this study are available from the corresponding author upon reasonable request.
